# Diabetes Mellitus

**DOI:** 10.1155/2014/108419

**Published:** 2014-01-23

**Authors:** Ilias Migdalis, David Leslie, Nikolaos Papanas, Paul Valensi, Helen Vlassara

**Affiliations:** ^1^2nd Medical Department and Diabetes Centre, NIMTS Hospital, 12 Monis Petraki, 11521 Athens, Greece; ^2^Department of Diabetes, Saint Bartholomew's Hospital, University of London and Bizard Institute, EC1A 7BE London, UK; ^3^Outpatient Clinic of the Diabetic Foot, Second Department of Internal Medicine, Democritus University of Thrace, 68100 Alexandroupolis, Greece; ^4^Department of Endocrinology, Diabetology and Nutrition, Jean Verdier Hospital, AP-HP, Paris Nord University, CRNH-IdF, CINFO, 93140 Bondy, France; ^5^Division of Experimental Diabetes and Aging, Mount Sinai School of Medicine, 1 Gustave L, Levy Place, P.O. Box 1460, New York City, NY 10029, USA

There is ongoing progress in the understanding, diagnosis, and management of diabetes mellitus [1]. The present special issue is devoted to the recent progress. There are 28 articles in total, which cover 6 thematic areas: epidemiology, pathogenesis, treatment, complications, nonalcoholic fatty liver (NAFL), and sleep disorders.

*Epidemiology.* In the context of the diabetes epidemic, W. Nseir et al. in their paper titled “*Seeking out high risk population: the prevalence characteristics and outcome of diabetic patients of Arab ethnicity hospitalized in internal medical and acute coronary units in Israel*” have looked at the prevalence of diabetes and issues relating to treatment efficacy among inpatients of hospitals with predominant Arab patients in Northern Israel. The prevalence of diabetes in this setting was 39% with a preponderance of women. Importantly, diabetic patients experienced prolonged hospitalisation, increased readmission rates, and suboptimal control. This data is important because it highlights the issues needing improvement.

*Pathogenesis.* M. Zhang et al. in their paper titled “*Fish and marine omega-3 polyunsatured fatty acid consumption and incidence of type 2 diabetes: a systematic review and meta-analysis*” have conducted a systematic review and meta-analysis on the relationship between the intake of fish and marine long-chain omega-3 polyunsaturated fatty acids and the incidence of type 2 diabetes mellitus (T2DM). They noted significant heterogeneity between included studies and could find no significant correlation between the effect of fish/seafood or marine polyunsaturated fatty acid consumption and risk of T2DM in general. However, oily fish polyunsaturated fatty acid, in particular, significantly reduced the risk of T2DM by 11% (*P* = 0.005). This work adds to the growing body of literature on the impact of polyunsaturated fatty acids on T2DM and attempts to take us beyond the conflicting results of the individual cohort studies [2, 3]. It now remains to scrutinise the selective beneficial effect of oily fish fatty acids by clinical trials with longer follow-up and determine any differences between high- and low-risk populations.

Turning their attention to type 1 diabetes (T1DM), K. Blaslov et al. in their paper titled “*Relationship between adiponectin level, insulin sensitivity, and metabolic syndrome in type 1 diabetic patients*” have explored insulin resistance and the metabolic syndrome in this condition. For this purpose, they have evaluated the relationship between adiponectin concentrations, metabolic syndrome, and insulin sensitivity. Adiponectin is known to exert a protective action against the metabolic syndrome, T2DM, and vascular injury [4]. At the same time, features of the metabolic syndrome may, albeit occasionally, be present in T1DM as well, while the role of adiponectin in this situation is less clear [5]. K. Blaslov et al. have now shown higher adiponectin levels to be associated with significantly lower waist circumference and serum glucose, along with a more favourable lipid profile. Logistic regression analysis confirmed adiponectin to bear a significant negative correlation with the metabolic syndrome (*P* = 0.014). Obviously, these results are promising and may have useful clinical implications. Nonetheless, it is rather premature to uphold a potential protective effect of adiponectin against development of metabolic syndrome in T1DM until further confirmation is obtained.

A. Al-Shukaili et al. in their paper titled “*Analysis of inflammatory mediators in type 2 diabetes patients*” have investigated cytokine levels and lymphocyte subsets in patients with T2DM. In comparison to healthy volunteers, T2DM patients exhibited significantly reduced interleukin (IL)-1*β*, IL-6, IL-15, and tumour necrosis factor-alpha (TNF-*α*), as well as significantly elevated IL-10, interferon-gamma (IFN-*γ*), and caspase-1, while lymphocyte subsets did not differ. Moreover, the presence of hypertension was linked with diminution of IL-1*β* and caspase-1. Overall, HbA_1c_ was positively correlated with IL-6 (*P* = 0.005), and body-mass index (BMI) was positively correlated with c-reactive protein (CRP) (*P* = 0.001) and TNF-*α*  (*P* = 0.013). These results confirm the activation of cytokines, primarily IL-6, in insulin resistance and T2DM [6, 7]. They also add new useful information on the contribution of hypertension to this inflammatory activity.

B. Liu et al. in their paper titled “*Ketosis-onset diabetes and ketosis-prone diabetes: same or not?*” have provided new knowledge on diabetes mellitus presenting with ketosis. This atypical form of diabetes, first described in Afro-Americans 25 years ago, is now known to present in two slightly different forms, that is, ketosis-prone diabetes and ketosis-onset diabetes [8]. In such patients, there may by considerable heterogeneity in terms of autoantibodies, which permits classification into 4 subgroups (*A* + *β* − , *A* − *β* + , *A* − *β*−, and *A* +  *β*+) [8]. B. Liu et al. now document that most patients with ketosis-prone diabetes had greater age and longer duration than ketosis-onset diabetes, although this does not apply to all subgroups. Moreover, in some of the subgroups, lower fasting plasma glucose and lower HbA_1c_ in ketosis-prone versus ketosis-onset diabetes was noted. The authors conclude that ketosis-prone and ketosis-onset diabetes are not uniformly the same in presentation. Clearly, there is a lot to learn about this rare form of diabetes in the future.

X.-F. Zhang et al. in their paper titled “*The ETS-domain transcription factor elk-1 regulates COX-2 gene expression and inhibits glucose-stimulated insulin secretion in the pancreatic β-cell line INS-1*” have used the pancreatic *β*-cell line INS-1 to study the association between the transcription factor Elk-1 and Cyclooxygenase-2 (COX-2). The rationale is that Elk-1 can significantly enhance the activation of COX-2 gene promoter, thereby impairing *β*-cell secretion via production of Prostaglandin E2 [9]. The authors report that Elk-1 can upregulate COX-2 expression and that excessive expression of the former can impair pancreatic glucose-stimulated insulin secretion. It may be hoped that these new findings can help towards defining new target molecules for potential future pharmaceutical intervention to hinder *β*-cell malfunction.

*Treatment.* Nowadays, the challenges in the management of T2DM arise not only from the abundance of different drugs and expert guidelines but also from the need for a modern patient-centred approach [10, 11]. In this issue, L. Chhabra et al. in their paper titled “*Challenges in the management of type 2 diabetes mellitus and cardiovascular risk factors in obese subjects: what is the evidence and what are the myths?*” discuss the goals, priorities, and fallacies in the treatment of T2DM (including improvement of cardiovascular risk factors) in obese subjects. They summarise recent evidence showing that intensive lifestyle modification does not succeed in reducing cardiovascular morbidity [12], but this does not negate the role of patient education on healthy dietary choices. They also emphasise the need to intensify antidiabetic treatment strategies in subjects with newly diagnosed T2DM, as these are most likely to revert to normoglycemia and gain a reduction in cardiovascular morbidity. Moreover, they maintain that stringent, as opposed to lenient, targets achieve more efficacious and durable weight loss, and they discuss the growing importance of bariatric surgery [13]. The latter can drastically improve serum glucose before actual weight loss and deserves to be considered not only in morbid but also in moderate obesity, at least in selected patients [13].

M. Haluzík et al. in their paper titled “*Renal effects of DPP-4 inhibitors: a focus on microalbuminuria*” have examined the effects of dipeptidyl peptidase-4 (DPP-4) inhibitors on the kidney, focusing on microalbuminuria. There is evidence to suggest that hyperglycemia, among other perturbations, interferes with glucagon-like peptide-1 (GLP-1) signalling in the kidney, which, in turn, may promote the expression of angiotensin II and TNF-*α*, thereby perpetuating glomerular injury [14]. In this context, DPP-4 inhibitors may contribute not only to the reduction of oxidative stress and inflammation but also to the improvement of endothelial dysfunction in the kidney, as discussed by the authors. It appears that their beneficial actions may be exerted by both GLP-1 dependent and GLP-1 independent mechanisms. Taken together, the data presented by M. Haluzík et al. cherish some hope that DPP-4 inhibitors may prove more useful in renal protection, while ongoing trials are anticipated to shed more light on this issue.

W.-H. Xiao et al. in their paper titled “*The effects of pioglitazone on biochemical markers of bone turnover in the patients with type 2 diabetes*” have investigated the potential untoward effects of pioglitazone on bone turnover in T2DM. They have demonstrated a significant reduction of procollagen type 1N-terminal propeptide (P1NP) and total alkaline phosphatase (BAP) in women but not in men, following 3 months of treatment. This effect was most pronounced in the post-rather than premenopausal group. Conversely, there was no change in osteocalcin and C-terminal telopeptide of type 1 collagen (CTX) levels in either gender. As already shown with thiazolidinediones [15], the present findings point to an adverse effect of pioglitazone on bone turnover. Specifically, this effect involves inhibition of bone formation but not of bone resorption, and postmenopausal women are most amenable. Clearly, these observations should find more application when choosing antidiabetic regimens in clinical practice, as it is now beginning to be appreciated in the era of individualised treatment [10].

A. Colatrella et al. in their paper titled “*Comparison of insulin lispro protamine suspension with NPH insulin in pregnant women with type 2 and gestational diabetes mellitus: maternal and perinatal outcomes*” have retrospectively compared maternal and perinatal outcomes of insulin lispro protamine suspension with neutral protamine Hagedorn (NPH) insulin in 25 pregnant women with T2DM and 64 with gestational diabetes (GDM). Insulin lispro protamine suspension is a relatively new formulation, which has hitherto been evaluated in T1DM and T2DM but not GDM [16]. More knowledge in GDM with this preparation is desirable, given the worldwide increase of GDM and the favourable outcomes achieved with detemir, another recent insulin analogue [17]. In the present analysis, there was no difference between the two treatment arms in terms of maternal outcomes (mode and time of delivery). Similarly, there was no difference in neonatal outcomes (e.g., newborn weight, neonatal hypoglycaemia rates, and congenital malformations) except for excessive ponderal index, which was more frequent with NPH. Moreover, fasting blood glucose, maternal hypoglycaemia rates, and weight gain did not differ between the two insulin regimens. Finally, insulin dose was lower with lispro protamine suspension. Thus, the new insulin formulation was not inferior to traditional NPH insulin.

J. Nicoll et al. in their paper titled “*Subetta treatment increases adiponectin secretion by mature human adipocytes in vitro*” have investigated the effect of Subetta (a novel composite preparation containing antibodies to the beta subunit of the insulin receptor and antibodies to endothelial nitric oxide synthase) on the production of adiponectin by human mature adipocytes. This was compared to rosiglitazone. It was found that Subetta significantly promoted adiponectin secretion. Although preliminary *in vitro *evidence only, the present results encourage further enquiry into the efficacy and the modes of action of Subetta as a potential emerging hypoglycaemic agent.

*Complications.* S. Pruhova et al. in their paper titled “*Chronic mild hyperglycemia in GCK-MODY patients does not increase carotid Intima-Media Thickness*” have examined carotid intima media thickness (CIMT) in patients with glucokinase-mutation maturity onset diabetes of the young (GCK-MODY or MODY2). In comparison to controls, subjects with GCK-MODY had insignificantly increased CIMT, adjusted for age, gender, and family status, while there were also no differences in frequency of myocardial infarction and ischaemic stroke between the two groups. The authors take this data as evidence for a low risk of developing macrovascular complications in GCK-MODY, despite chronic mild hyperglycaemia. However, caution is needed to avoid physician negligence in pursuing glycaemic targets in such patients.

M. Cetin et al. in their paper titled “*Relation of epicardial fat thickness with carotid intima-media thickness in patients with type 2 diabetes mellitus*” have examined the association of CIMT with epicardial fat thickness, an emerging potential additional indicator of cardiovascular risk [18], in T2DM. Both parameters were increased in T2DM patients versus controls. In T2DM, epicardial fat thickness was correlated with CIMT, waist circumference, BMI, age, and diabetes duration. In linear regression analysis, CIMT and waist circumference emerged as independent predictors of epicardial fat thickness. Interestingly, the authors defined a 6.3 mm cut-off for epicardial fat thickness, which yielded 72.5% sensitivity and 71.7% specificity for the diagnosis of increased CIMT denoting incipient atherosclerosis. This work adds to the growing evidence that epicardial fat thickness may prove useful as a marker of cardiovascular risk, and further experience is now awaited.

Y. Zhang et al. in their paper titled “*Assessment of carotid atherosclerosis in type 2 diabetes mellitus patients with microalbuminuria by high-frequency ultrasonography*” present data from high-frequency ultrasound employed in the assessment of CIMT and atherosclerotic plaques in T2DM patients with micro-versus those with normoalbuminuria. IMT was predictably higher in the former. Overall, IMT exhibited significant correlations with urinary albumin excretion rate, age, diabetes duration, HbA_1c_, waist circumference, BMI, and systolic blood pressure. Importantly, urinary albumin excretion rate was among the independent predictors of CIMT.

Analysing insulin sensitivity and antioxidant enzyme activity in patients with or without T2DM and various stroke subtypes, A. Jotic et al. in their paper titled “*Type 2 diabetic patients with ischemic stroke: decreased insulin sensitivity and decreases in antioxidant enzyme activity are related to different stroke subtypes*” show that reduced insulin sensitivity and glutathione reductase are associated with atherothrombotic and lacunar stroke in T2DM. These results open up a new vista linking insulin resistance with stroke via diminished antioxidant enzyme activity, but the attractive question why this particularly applies to some kinds of stroke remains unanswered.

Wound healing may be impaired in diabetes, especially in the foot [19]. C. Shrestha et al. in their paper titled “*Enhanced healing of diabetic wounds by subcutaneous administration of human umbilical cord derived stem cells and their conditioned media*” have used mesenchymal stem cells from the umbilical cord and their conditioned media to promote wound closure in the experimental model (dorsal wound in db/db mice). Phosphate buffer solution was used as control. Both stem cells and their conditioned media accelerated healing (especially the latter). The results are encouraging and further clinical experience is desirable.

Going from the experimental setting to humans, diabetic foot infections increase the risk of lower-limb amputations [20]. Tissue cultures are the gold standard for the identification of true pathogens [21]. Using these, M. Demetriou et al. in their paper titled “*Determinants of microbial load in infected diabetic foot ulcers: a pilot study*” have attempted to define parameters predicting a high microbial load in diabetic foot infections. The number of isolates on tissue cultures and white blood cell count were found as the most powerful predictors of microbial load. Other predictors included platelet count and clinical severity of infection. The authors suggest that high blood cell and platelet count, as well as a sinister clinical manifestation, call for a more aggressive initial antibiotic regimen to cover a diversity of pathogens, and this sounds quite reasonable.

Polyneuropathy is a cardinal aetiological factor in the pathogenesis of the diabetic foot [19, 20], and its early diagnosis with accurate clinical tests is of utmost importance [22]. T. Mete et al. in their paper titled “*Comparison of efficiencies of Michigan neuropathy screening instrument, neurothesiometer, and electromyography for diagnosis of diabetic neuropathy*” have used the Michigan Neuropathy Screening Instrument (MNSI), the neurothesiometer, and nerve conduction study (NCS) to detect diabetic polyneuropathy. The neurothesiometer and NCS yielded higher rates of polyneuropathy (74.5% and 46.2%, resp.), as compared to clinical examination by MNSI (32.1%). The authors use these findings as argument that neurothesiometer and NCS should be employed to increase timely detection of polyneuropathy.

P. Thomakos et al. in their paper titled “*Cigarette smoking is associated with prolongation of the QTc interval duration in patients with type 2 diabetes mellitus*” have assessed the effect of smoking on autonomic nerve function and QTc interval in T2DM. They could demonstrate significant prolongation of the QTc interval during both day and night in smokers versus nonsmokers. Conversely, there was no difference in attributes of autonomic nerve function between the two groups. It was concluded that smoking prolonged the QTc interval by mechanism(s) independent of autonomic dysfunction. Whether this effect contributes to increased cardiovascular risk remains to be further queried.

S. Meguro et al. in their paper titled “*Past obesity as well as present body weight status is a risk factor for diabetic nephropathy*” have sought the association between prevalence of nephropathy and past obesity status in a large series of Japanese patients with T2DM. Nephropathy was significantly (*P* < 0.017) more frequent in the event of prior or current obesity than in constantly normal-weight subjects. Both past and present obesity belonged to the independent predictors of neuropathy in logistic regression analysis. These observations are important, given the increasing appreciation of an obesity-related nephropathy, and it would be highly welcome to have studies providing more information on duration of obesity, fluctuations of BMI, and the effect of anti-diabetic treatment and lifestyle changes on the development and/or evolution of diabetic kidney disease.

N. Grandfils et al. in their paper titled “*Glucose lowering therapeutic strategies for type 2 diabetic patients with chronic kidney disease in primary care setting in France: a cross-sectional study*” have scrutinised the strategies and priorities used by French general practitioners when deciding on anti-diabetic treatment for T2DM patients with moderate/severe chronic kidney disease. Perceived severity of diabetes, rather than of kidney disease, was the most important factor in choosing treatment. Of note, most practitioners tended to underestimate the risk of hypoglycaemia in this vulnerable population. In pursuit of stringent glycaemic targets, 2/3 of patients received drugs not safe enough for use in moderate/severe kidney disease. This observational study highlights the importance of modern individualised treatment, based on comorbidities, life expectancy, diabetes duration, hypoglycaemia awareness, and other patient parameters [10, 11]. Clearly, we need to improve physicians' understanding of these intricate issues.

B. Xu et al. in their paper titled “*Low serum magnesium level is associated with microalbuminuria in Chinese diabetic patients*” have addressed the relationship between low serum magnesium levels and microalbuminuria in Chinese subjects with diabetes mellitus. Microalbuminuria was significantly (*P* < 0.0001) more frequent in subjects with low serum magnesium. Even after adjustment for several covariates, there was an almost twofold increase in the frequency of microalbuminuria among those with the lowest magnesium levels. Given that magnesium harbours antioxidant actions [23], an arguable explanation may be that low magnesium contributes to oxidative stress, which, in turn, increases kidney damage [24]. We should, however, bear in mind that dietary habits and the effect of diuretics may be confounding factors, and so further experience regarding the aforementioned association is needed.

V. Jegdic et al. in their paper titled “*Physical fitness in children with type 1 diabetes measured with six-minute walk test*” have addressed the question whether children with T1DM exhibit lower physical fitness and whether, should this be the case, increased HbA_1c_ plays a contributory role. By means of the 6-minute walk test, they demonstrated lower physical fitness in T1DM, but this did not appear to be dependent on HbA_1c_. This rather surprising result calls for increased medical attention offered to T1DM children and, therefore, merits prompt replication in other populations.

*NAFL.* A. N. Mavrogiannaki and I. N. Migdalis in their paper titled “*Nonalcoholic Fatty liver disease, diabetes mellitus and cardiovascular disease: newer data*” present new information on NAFL, T2DM, and cardiovascular disease. They review the evidence on the prevalence of NAFL in T2DM (and, more rarely, in T1DM) and on the prevalence of cardiovascular morbidity in subjects with NAFL. Pathogenesis of NAFL mainly involves insulin resistance. New imaging techniques (such as elastography), emergent breath tests, and new biomarkers represent the progress that is being achieved in diagnosis. Weight loss, antioxidant drugs, and hypolipidaemic agents have been used in the management. As regards anti-diabetic treatment, some favourable outcomes have been achieved with metformin, thiazolidinediones, and GLP-1 analogues [25], but there is no uniformly accepted therapeutic modality.

Following on, E. Bacchi and P. Moghetti in their paper titled “*Exercise for hepatic fat accumulation in type 2 diabetic subjects*” discuss the role of exercise in the management of NAFL in T2DM. Indeed, there is interesting accumulating data that exercise can, independently of dietary changes, contribute to the reduction of hepatic steatosis. Of relevance, this appears to hold true both for aerobic and for anaerobic exercise. However, the magnitude of achieved effects is quite variable, and the precise effect on liver histology need further examination.

T. Hirata et al. in their paper titled “*Effect of telmisartan or losartan for treatment of nonalcoholic fatty liver disease: fatty liver protection trial by telmisartan or losartan study (FANTASY)*” have examined the efficacy of losartan and telmisartan in improving NAFL in the presence of hypertension and T2DM. Telmisartan-treated patients exhibited significant reductions in serum-free fatty acids and liver fat (as evaluated by Computed Tomography). These promising results may be ascribed to the unique activation of peroxisome proliferator-activated receptors gamma (PPAR-*γ*) by telmisartan [26] and call for replication in larger trials and different ethnic groups. If established, this action of telmisartan will improve our therapeutic interventions for NAFL.

*Sleep Disorders.* Sleep disorders are now increasingly being appreciated in diabetes and prediabetes. Indeed, somnolence, obstructive sleep apnoea, and sleep deprivation are now frequently examined in T2DM patients [27, 28]. W.A.Wan Mahmood et al. in their paper titled “*Association between sleep disruption and levels of lipids in Caucasians with type 2 diabetes*” have examined lipid profile in T2DM patients with poor sleep quality. They have shown elevated total cholesterol in subjects with long sleep duration and elevated triglycerides in those with short sleep duration. Sleep duration and quality were identified as major contributors to adverse serum lipids. This study adds to our knowledge on the disadvantageous effect of sleep disorders on serum lipids, as already shown for obstructive sleep apnoea [29].

J. Liu et al. in their paper titled “*The association of sleep disorder, obesity status, and diabetes mellitus among US adults—the NHANES 2009-2010 survey results*” have used the National Health and Nutrition Examination Survey (NHANES) 2009-2010 data to examine the relationship of sleep disorders with T2DM. In summary, after adjustment for several covariates including BMI, they have found that sleep disorders increase the risk of diabetes by 38%. Importantly, most of this increased risk is driven through subjects' obesity. These observations come from a large robust database and consolidate our knowledge pertaining to sleep perturbations in diabetes.

*Conclusions.* There is ongoing progress in diabetes research and care, as exemplified in the areas covered in this special issue. Several other fields are also showing progress, such as management of the diabetic foot [30], but these are not discussed in the present issue. From a clinical perspective, it is now important to integrate this progress into clinical reality, and this remains an ongoing challenge.

*Ilias***  ***Migdalis* *Ilias***  ***Migdalis* *Helen Vlassara* *Helen Vlassara* *David Leslie* *David Leslie* *Nikolaos Papanas* *Nikolaos Papanas* *Paul Valensi* *Paul Valensi*
